# Polydatin Attenuates H_2_O_2_-Induced Oxidative Stress via PKC Pathway

**DOI:** 10.1155/2016/5139458

**Published:** 2016-01-04

**Authors:** Huilian Qiao, Hao Chen, Yuhang Dong, He Ma, Guangchao Zhao, Fakuan Tang, Zhen Li

**Affiliations:** ^1^Department of Histology and Embryology, The Fourth Military Medical University, Xi'an 710032, China; ^2^Department of Cardiovascular Center, The 309th Hospital of Chinese PLA, Beijing 100193, China

## Abstract

Oxidative stress plays an important role in the pathogenesis of endothelial dysfunction, which is found to precede the development of diverse cardiovascular diseases (CVDs). The aim of this study was to observe the protective effects of PD against H_2_O_2_-induced oxidative stress injury (OSI) in human umbilical vein endothelial cells (HUVECs) and the possible mechanism of PD in OSI treatment. HUVECs were subjected to H_2_O_2_ in the absence or presence of PD. It turned out that PD improved cell viability and adhesive and migratory abilities, inhibited the release of lactate dehydrogenase (LDH) and reactive oxygen species (ROS), and elevated the content of glutathione peroxidase (GSH-Px) and superoxide dismutase (SOD). TUNEL, fluorometric assays, and Western blotting showed that OSI upregulated the apoptosis ratio, the activity of caspase-3 and the level of proapoptotic protein Bax and decreased the level of antiapoptotic protein Bcl-2. However, PD treatment partially reversed these damage effects and Protein Kinase C (PKC) activation by thymeleatoxin (THX) in turn eliminated the antiapoptotic effect of PD. Furthermore, PD attenuated the H_2_O_2_-induced phosphorylation of PKCs *α* and *δ* and increased the phosphorylation of PKC *ε*. Our results indicated that PD might exert protective effects against OSI through various interactions with PKC pathway.

## 1. Introduction

Endothelial cells maintain vascular homeostasis through inhibiting platelet aggregation, preventing adhesion of leukocytes, and limiting proliferation of vascular smooth muscle and through balancing vasodilators and vasoconstrictors to manipulate vascular tone. Endothelial dysfunction is reported to be the initial step of many cardiovascular pathophysiological progresses, such as hypertension [1], coronary artery disease [2], or heart failure [3]. Meanwhile, clinical intervention to reserve or augment the endothelium function is recommended to be an appropriated method to CVD treatments. The latest studies show that oxidative stress plays a main character in the pathogenesis of endothelial dysfunction [4], and oxidative stress induces apoptosis [5].

Polydatin, 3,4′,5-trihydroxystilbene-3-*β*-mono-d-glucoside, is extracted from the root stem of a traditional Chinese herbal medicine named* Polygonum cuspidatum *Sieb. A large number of evidences show that PD is involved in a wide range of biological functions, such as antiplatelet aggregation [6] and antiatherosclerosis [7]. It has been demonstrated that PD acts as antioxidant agents to prevent severe diseases. Particularly the PD induced antioxidative activity is reported to reverse renal injury by attenuating oxidative stress-related inflammatory responses in fructose-induced urate nephropathic [8]. Besides, PD could eliminate ischemia reperfusion (I/R) damage toward brain [9] and lung [10], in which the antioxidative stress ability was thought to be one of the key regulators. PD is proved to exert inhibitory effects on I/R-induced apoptosis in myocardium [11]. Resveratrol (RSV), the deglycosylation form of (PD), could mitigate oxidative stress-induced apoptosis in C2C12 cells and primary neonatal rat cardiomyocytes [12] and increase viability of dopaminergic neurons against neurotoxicity [13]. PKCs are involved in the effects of PD on cardiac ischemia [14] and hypoxic pulmonary hypertension [15]. However, whether PKC cellular circuit is involved in the prevention by PD from H_2_O_2_-induced OSI has not been elucidated to date.

The Protein Kinase C (PKC) is a group of serine/threonine kinases and important intracellular signal transduction molecules. It possesses multiple functions in regulating cellular differentiation [16], proliferation [17], and apoptosis [18]. PKCs are particularly important in redox reaction because their regions are susceptible to redox modifications, since variations of redox state have consequences on their activity [19]. According to the N-terminal regulatory domains, PKCs are classified into three subfamilies: conventional (*α*, *β*I, *β*II, and *γ*), novel (*δ*, *ε*, *η*, *θ*, and *μ*), and atypical (*ζ* and *λ*). The expression of PKCs in cardiac tissue differs with species and cell type, but most adult mammals express PKC *α*, *β*, *δ*, and *ε* [20]. PKC *α*, the first found conventional PKC, is expressed at a high level in cardiovascular system and is activated in cardiomyocytes subjected to oxidative stress [21]. PKC *δ* is the representative of novel PKC and is widely involved in oxidative stress events [22]. Although PKCs *δ* and *ε* are highly homologous, PKC *ε* is known for its antioxidative ability [23] and cardioprotection in the I/R model [24] which differs from the function of PKC *δ*.

In this study, we investigated the role of PD in H_2_O_2_-induced OSI of HUVECs. Then we explored whether PD protected HUVECs against OSI injury via regulation of PKC signaling pathway. Furthermore the possible involvement of PKCs *α*, *δ*, and *ε*, the representative and unique isoforms, was demonstrated in PD treatment of the oxidative stress injured HUVECs.

## 2. Materials and Methods

### 2.1. Chemicals and Reagents

The PD was purchased from WeiJia Technology Company (Xi'an, China), and MTT [3-(4,5-dimethylthiazol-2-yl)-2,5-diphenyltetrazolium bromide], PKC pathway activator thymeleatoxin (THX), and 2′,7′-dichlorofluorescein diacetate (2′,7′-DCFH-DA) were purchased from Sigma-Aldrich Company (St. Louis, MO, USA). Terminal deoxynucleotidyl transferase dUTP nick-end labeling (TUNEL) kits were purchased from Roche Company (Mannheim, Germany). The kits for the detection of LDH, GSH-Px, and SOD concentrations and caspase-3 activity were purchased from the Institute of Jiancheng Bioengineering (Nanjing, Jiangsu, China). The PKC *α*, PKC *δ*, PKC *ε*, Bcl-2, Bax, and GAPDH antibodies were purchased from Bioss (Beijing, China). The p-PKC *α*/*β* (Thr638/641) and p-PKC *δ* (Tyr311) antibodies were purchased from Proteintech Group (Chicago, USA) and the p-PKC *ε* (Ser729) antibody was purchased from Sigma-Aldrich Company (St. Louis, MO, USA). The secondary antibodies (goat anti-rabbit and anti-mouse) were purchased from Zhongshan Company (Beijing, China).

### 2.2. Cell Culture and Treatments

HUVECs (ATCC CRL-1730; Shanghai Tiancheng Technology Company) were cultured in RPMI 1640 medium (Hyclone, Utah, USA) containing heat-inactivated 10% fetal bovine serum, 100 U/mL penicillin, and 100 *μ*g/mL (100 U/mL) streptomycin at 37°C in 5% CO_2_. The PD stock solution was prepared in 1% dimethylsulfoxide (DMSO) and diluted with culture medium immediately prior to the experiment. The cells were treated with H_2_O_2_ (400 *μ*M) [25] alone or in combination with PD for 4 h. After the treatments, the cells were prepared for further studies.

### 2.3. Analysis of Cell Viability and Morphology

The viability of HUVECs was assessed using the MTT assay referring to a previous study [25]. Briefly, cells were seeded in 96-well plates. After being treated with H_2_O_2_ and PD of different concentrations for 4 h, the cells were washed for 3 times with PBS, then 100 *μ*L of MTT solution (0.5 mg/mL) was added directly to the culture medium, and the cells were incubated for 4 h at 37°C. After MTT removal, 100 *μ*L of N,N-dimethylformamide was added to each well to dissolve the formazan crystals, and the plates were shaken for 15 min at 37°C. Absorbance of samples was measured at 490 nm using a Microplate Reader (SpectraMax 190, Molecular Device, USA), and the results were expressed as % cell viability by the analysis of an OD value.

### 2.4. Cell Adhesion Assay

Cell adhesion test was assessed as previously described [26]. After different treatments, cells (1 × 10^4^ cell per well) were resuspended in culture medium, placed on fibronectin-coated 6-well plates, and incubated for 30 min at 37°C. Then the cells were gently washed with PBS for 3 times. The adherent cells were counted by independent and blinded investigators. The results were expressed as the percentage of control.

### 2.5. Wound Healing Assay

As previously described [27], HUVECs were seeded in 6-well plates and cultured for 24 h. Subsequently, we scratched confluent cell monolayers with a P200 pipette tip to make three parallel “wounds” in each well and washed the cells with serum-free medium. After the cells were incubated with different treatments for 8 h, the migrated cells were photographed using an inverted/phase-contrast microscopy (Olympus BX61, Japan). The mean distance between the two ends of the scratch was quantified by manual measurements.

### 2.6. Measurement of the Content of ROS

Measurement of intracellular ROS was based on ROS-mediated conversion of nonfluorescent 2′,7′-DCFH-DA into fluorescent DCFH, as previously described [25]. After being seeded and incubated with different treatments for 4 h in black 96-well plates, the cells were washed with PBS and subsequently incubated with DCFH-DA (20 microM) in PBS at 37°C for 2 h. At the end of incubation, the fluorescence of the cells was measured using an FLX 800 microplate fluorescence reader (the emission wavelength, 530 nm; the excitation wavelength, 485 nm) (Biotech Instruments Inc., USA). The background was from cell-free conditions. The results were expressed as the fold of the fluorescence intensity compared to the control group.

### 2.7. Measurement of LDH Release

LDH release, which could reflect the cell membrane integrity, was detected with an assay kit (A020-1, Jiancheng, Nanjing, China) according to the manufacturer's instructions. Briefly, after incubation with different treatments for 4 h, the cells were centrifuged at 300 ×g for 10 min. Then 60 *μ*L supernatant was added with 30 *μ*L LDH substrate solution for incubation for 30 min at 37°C. The activity of enzyme was expressed as units per liter, and the absorbance was read at 440 nm.

### 2.8. Measurement of the Intracellular Contents of GSH-Px and SOD

Commercially available kits for the measurement of SOD (A001-3, Jiancheng, Nanjing) and GSH-Px (A061-2, Jiancheng, Nanjing, China) were used according to the manufacturer's instructions. After being lysed by the compound lysis buffer and centrifuged at 12000 r/mim for 5 min, the cell supernatant was collected for detecting the contents of GSH and SOD that were expressed as units/mg protein. One unit of SOD was equal to the amount that reduced the absorbance by a half at 550 nm. The GSH-Px activity assay was performed by quantifying the oxidation rate of reduced GSH to oxidized form by H_2_O_2_ and catalyzed by GSH-Px. One unit of GSH-Px was equal to the amount that reduced the level of GSH at 412 nm by 1 mM in one min per mg protein.

### 2.9. Cellular Apoptosis Assay

The apoptosis of HUVECs was analyzed by performing a TUNEL assay using a commercial kit (11684817910, Roche Company, Germany) according to the manufacturer's instructions. After being fixed in paraformaldehyde (4%) for 24 h, the nuclei of the apoptotic cells were stained green, and all of the nuclei were stained blue with 4′,6-diamino-2-phenylindole (DAPI). The TUNEL positive ratio was expressed as the ratio of positively stained cells to the total number of HUVECs.

### 2.10. Measurement of Caspase-3 Activity

Apoptotic cell death was determined by caspase-3 activation via a fluorometric kit (G007, Jiancheng, Nanjing, China). Briefly, after being seeded and treated in 96-well plates, endothelial cells were harvested using caspase lysis buffers (50 mM HEPES, pH 7.4, 0.1% Chaps, 5 mM dithiothreitol, 0.1 mM EDTA, and 0.1% Triton X-100) for 5 min on ice and centrifuged at 12000 rpm for 10 min at 4°C. Then the supernatant (50 *μ*g) was saved and incubated with 10 *μ*L caspase-3 substrate (Ac-DEVDpNA) for 1 h at 37°C. The fluorescence emission was measured using a Biotech microplate fluorescence reader at an excitation wavelength of 400 nm (emission wavelength, 505 nm). The fluorescence intensity was set as 100% in the control group.

### 2.11. Western Blot Assay

HUVECs were washed with PBS and lysed with 100 *μ*L RIPA Lysis buffer (Beyotime, Shanghai, China, P0013C) containing 50 mM Tris-HCl (pH 7.4), 150 mM NaCl, 1% NP-40, and 0.1% SDS for 30 min on ice. Then cells were scraped and centrifuged for 10 min at 4°C and the concentration was qualified with a BCA protein Assay Kit (Lot#PI208677, Thermo, Rochford, USA). Proteins of a total content of 50 *μ*g were separated on SDS-polyacrylamide gel electrophoresis gels (SDS-PAGE) and transferred to nitrocellulose membranes. The membranes were blocked with 5% nonfat milk and then incubated with primary antibodies (PKC *δ* and PKC *ε* with a dilution of 1 : 2000, PKC *α*, p-PKC *ε*, Bcl-2, Bax and GAPDH with a dilution of 1 : 1000, p-PKC *α*/*β* with a dilution of 1 : 300, and p-PKC *δ* with a dilution of 1 : 100) and horseradish peroxidase-conjugated secondary antibody (Zhongshan Jinqiao, Beijing). The blot was developed with a Supersignal Chemiluminescence detection kit (Solarbio, Beijing) and observed with ChemiDoc XRS+ Molecular Imager (BIO-RAD).

### 2.12. Statistical Analysis

All values in the text and figures are presented as mean ± SEM of at least three independent experiments. Analysis of variance of parametric difference among experimental data was performed by one-way ANOVA and Dunnett's test was then performed for multiple comparisons [16]. Western blot densities were analyzed with Kruskal-Wallis ANOVA test [28]. Values of *p* < 0.05 were considered statistically significant.

## 3. Results

### 3.1. Effects of PD on the Cell Viability, Adhesive Ability, and Migratory Ability of OS-Injured HUVECs

**Figure 1:**
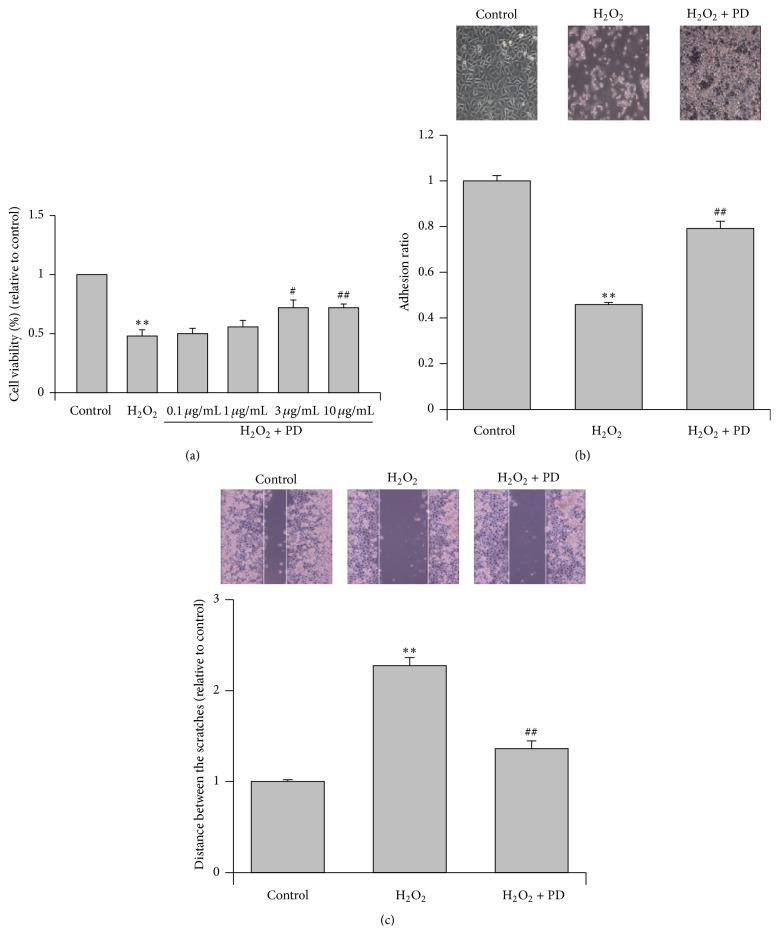
Effects of PD on the viability, adhesive ability, and migratory ability of OS-injured HUVECs. (a) HUVECs were subjected to H_2_O_2_ (400 *μ*M) in the absence or presence of PD of different concentrations for 4 hours. The cell viability of HUVECs was measured by the MTT assay. (b) The adhesive ability of the HUVECs was assessed by performing an adhesion assay, and cell adhesion was expressed as an adhesion ratio. The number of adherent cells in the control group was set at 100%. The cell morphology was observed using inverted/phase-contrast microscopy, and images were obtained. (c) The migratory ability of the HUVECs was assessed by performing a wound healing assay, and the migratory ability was expressed as the mean distance between the two ends of the scratch. The mean distance in the control group was set at 100%. The results are expressed as mean ± SEM, *n* = 3 (^*∗∗*^ *p* < 0.01, compared to the control group, ^#^ *p* < 0.05, ^##^ *p* < 0.01, compared to the H_2_O_2_ group). Significant differences between groups were analyzed with one-way ANOVA.

To investigate whether PD could prevent endothelium from OSI, HUVECs were subjected to H_2_O_2_ (400 *μ*M) in the absence or presence of PD of various concentrations (0.1–10 *μ*g/mL) for 4 hours. The cell viability decreased to 48% after H_2_O_2_ treatment. Although the difference between H_2_O_2_ group and the low dose group (0.1 *μ*g/mL) was not manifest, the cell viability elevated to 72% when the concentration of PD was 3 *μ*g/mL or higher (Figure 1(a)). Based on these results, treatment with 400 *μ*M H_2_O_2_ and 3 *μ*g/mL PD for 4 hours was selected for the further experiments. As shown in Figure 1(b), H_2_O_2_ treatment resulted in the change of cell morphology, such as remarkable cell shrinkage and reduced refraction. Meanwhile, the cellular adhesion rate in the H_2_O_2_ group was reduced by more than 50% compared to the control group. The PD treatment significantly improved the cellular adhesion rate but the cell shape did not evidently recover compared to the H_2_O_2_ group. As demonstrated in Figure 1(c), the distance between the scratches increased significantly after treatment with H_2_O_2_ compared to the control group but curtailed almost 40% after PD treatment compared to the H_2_O_2_ group, indicating that PD enhanced the migratory ability of HUVECs injured by H_2_O_2_.

### 3.2. Effects of PD on LDH, ROS, GSH-Px, and SOD Measured in OS-Injured HUVECs

**Figure 2:**
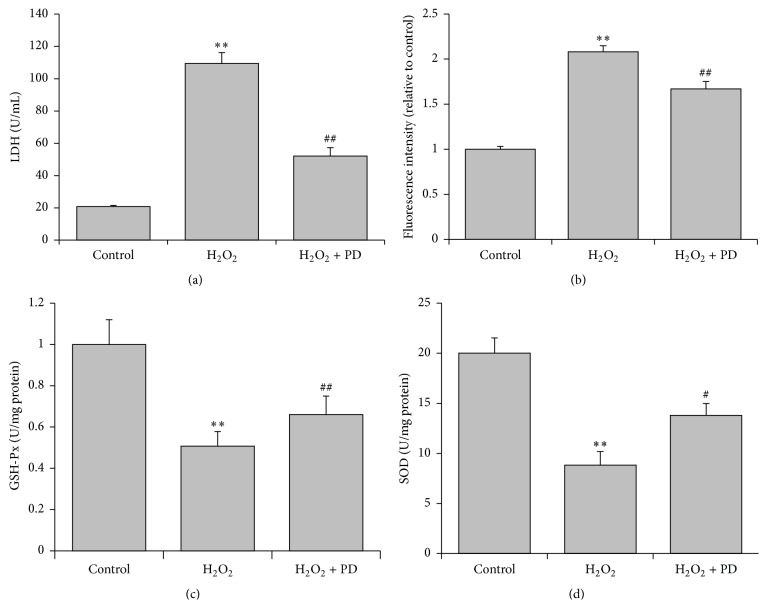
Effects of PD on LDH, ROS, GSH-Px, and SOD in OS-injured HUVECs. HUVECs were treated with H_2_O_2_ (400 *μ*M) alone or with PD (3 *μ*g/mL). (a) The release of LDH from HUVECs was detected using an assay kit. (b) The intensity of DCFH fluorescence was measured to evaluate the intracellular ROS level. (c) The intracellular GSH-Px level of HUVECs. (d) The intracellular SOD level of HUVECs. The results are expressed as mean ± SEM, *n* = 3 (^*∗∗*^ *p* < 0.01, compared to control group, ^#^ *p* < 0.05, ^##^ *p* < 0.01, compared to H_2_O_2_ group). Significant differences between groups were analyzed with one-way ANOVA.

To explore the effects of PD on the oxidative stress of H_2_O_2_-injured HUVECs, we next measured the levels of intracellular LDH, ROS, GSH-Px, and SOD. H_2_O_2_ treatment elevated the level of LDH to 5 times (Figure 2(a)), doubled ROS release (Figure 2(b)), and reduced the content of GSH-Px and SOD by a half (Figures 2(c) and 2(d)) compared to the control group. However, PD replenishment induced a remarkable decrease in the level of LDH by more than 50% (Figure 2(a)) and ROS by 18% and significantly attenuated the changes in the content of GSH-Px by 33% (Figure 2(c)) and SOD by 60% (Figure 2(d)) compared to the H_2_O_2_ group, indicating the protective activity of PD.

### 3.3. Effects of PD and THX on the Apoptosis of OS-Injured HUVECs

**Figure 3:**
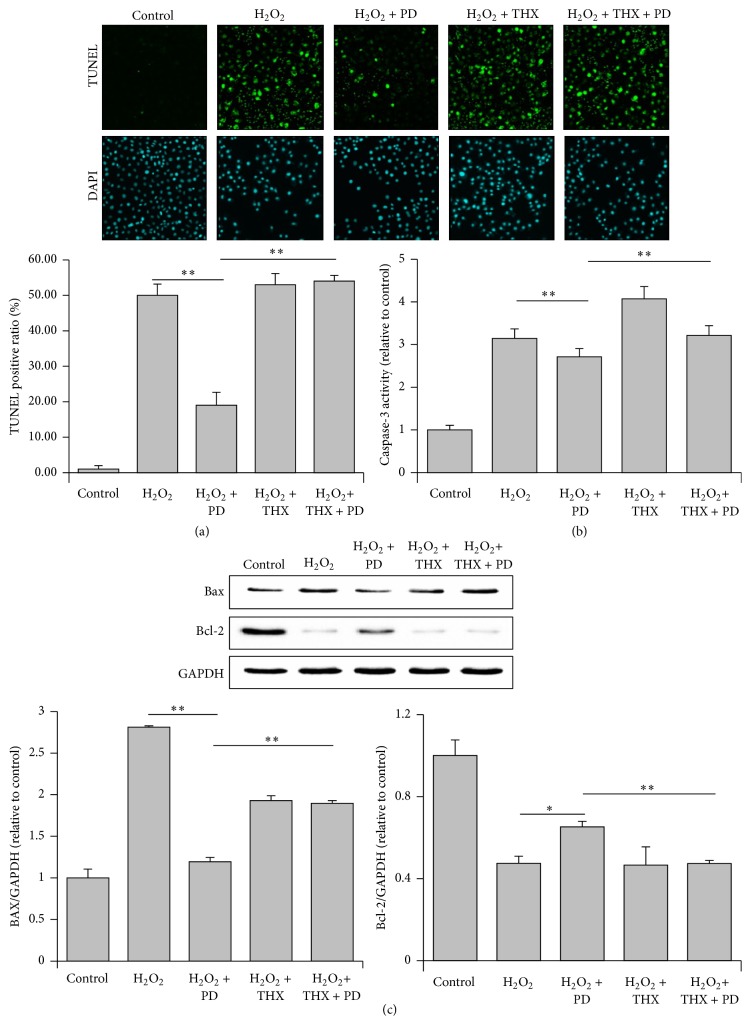
Effects of PD and THX on the apoptosis of OS-injured HUVECs. (a) The apoptosis of the HUVECs was assessed by the TUNEL assay. TUNEL staining was performed to stain the nuclei of the apoptotic cells (green), and DAPI was used to stain all of the nuclei (blue). The apoptotic index was expressed as the percentage of positively stained apoptotic cells out of the total number of cells counted. (b) The caspase-3 activity was measured via a fluorometric kit. (c) The Western blots images of Bcl-2 and Bax are, respectively, shown in the upper panel. The lower panels represent the densitometric analysis of the data. GAPDH was used as a loading control. The results are expressed as mean ± SEM, *n* = 3 (^*∗*^ *p* < 0.05, ^*∗∗*^ *p* < 0.01). Significant differences between groups were analyzed with one-way ANOVA.

The antiapoptotic effect of PD has been demonstrated in previous studies [11, 12]. We therefore explored whether PD could attenuate the apoptosis of H_2_O_2_-injured HUVECs and the possible involvement of PKC signaling. As shown in Figure 3(a), the TUNEL positive ratio increased from less than 1% to 50% after H_2_O_2_ treatment and decreased to 25% when PD was replenished. Furthermore, the activity of caspase-3 increased by 200% after H_2_O_2_ treatment compared to the control group and decreased by almost 30% when PD was added (Figure 3(b)). Nonetheless, after the forced activation of PKC by THX (100 nM) [29], the apoptosis ratio and caspase-3 activity increased to a degree even higher than those of the H_2_O_2_-treated group (Figures 3(a) and 3(b)). In line with this observation, the attenuated expression of proapoptotic molecule Bax and the corresponding enhanced expression of antiapoptotic signal Bcl-2 were noticeable when the cells were treated with H_2_O_2_ and PD compared to the H_2_O_2_ group, whereas THX treatment suppressed the effects of PD on Bax and Bcl-2 expression (Figure 3(c)).

### 3.4. Effects of PD on Phosphorylation Level of PKC in OS-Injured HUVECs

**Figure 4:**
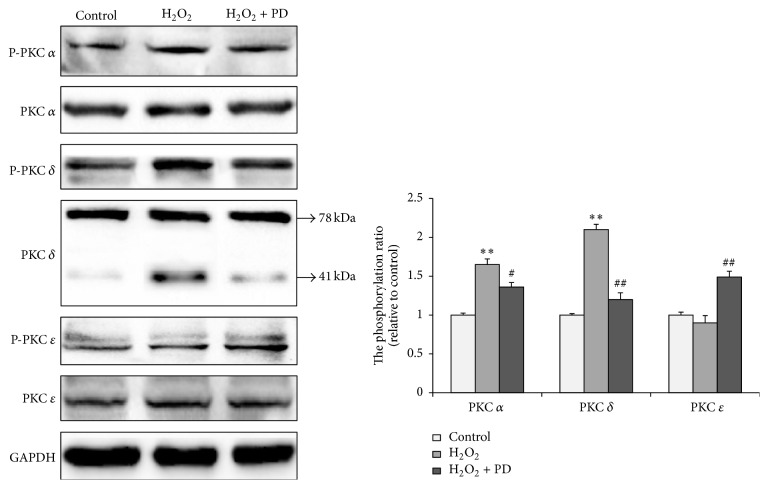
Effects of PD on the levels of p-PKC *δ* of OS-injured HUVECs. The Western blots images of p-PKC *α*, p-PKC *δ*, p-PKC *ε*, and total PKCs *α*, *δ*, and *ε* are shown in the left panel. The right panel represents the densitometric analysis of the data. Expression of each p-PKC was normalized to total PKC content, and GAPDH was used as a loading control. The result are expressed as mean ± SEM, *n* = 3 (^*∗∗*^ *p* < 0.01, compared to the control group, ^#^ *p* < 0.05, ^##^ *p* < 0.01, compared to the H_2_O_2_ group). Significant differences between groups were analyzed with one-way ANOVA.

Our above data collectively raise the possibility that PD may exert its antiapoptotic effect on endothelial cells through PKC dependent pathway. Since PKC *α* and PKC *δ* have been reported to be activated by oxidative stress [30] and PKC *ε* is an important molecule in the protection of OSI [24], we determined whether PD attenuates OSI in HUVECs through regulation of these three PKCs. As shown in the left panel of Figure 4, HUVECs expressed all the three isoforms of PKC. No obvious differences in the total amount of PKCs *α*, *δ*, and *ε* were observed among the three groups as indicated in the left panel. It is worth noting that the level of proteolytic fragment of PKC *δ* (41 kDa) significantly increased along with H_2_O_2_ stimulation (<0.01) but abated remarkably after PD incubation (<0.05). A ratio of phospho-PKC to the total PKC was calculated to evaluate the activation of PKC upon stimulation to oxidative stress (the right panel of Figure 4). An increased phosphorylation level of both PKC *α* (by 65%) and PKC *δ* (by 100%) was observed after H_2_O_2_ treatment, while PD replenishment was able to decrease the phosphorylation level of PKC *α* (by 17.6%) and PKC *δ* (by 42.8%) compared to the H_2_O_2_ group. As to the content of p-PKC *ε*, little difference was found between the control group and H_2_O_2_ single treated group (>0.05). But compared with H_2_O_2_ group, the level of p-PKC *ε* was increased by 50.5% after being treated in combination with PD compared to H_2_O_2_ single treated group.

## 4. Discussion

It is well documented that endothelial dysfunction is a crucial event in the development of CVDs including atherosclerosis, myocardial ischemia reperfusion, and hypertension. The endothelial cells are very sensitive to OSI, which is involved in endothelial dysfunction [31]. In the present study, we established H_2_O_2_-induced OSI model to study the protective effect of PD against endothelial damage. We confirmed that PD conferred protection to HUVECs against H_2_O_2_ by improving cell viability and adhesive and migratory abilities. These findings concur with previous studies where resveratrol, the deglycosylation form of PD, significantly attenuates the decrease of cell viability in OS injured HUVECs [32] and the methamphetamine-induced neurotoxicity in mouse mesencephalic neurons [13] and evidently enhance activities of proliferation, adhesion, and migration of endothelial progenitor cells [33]. Thus, our results suggest that supplementation with PD could ameliorate endothelial dysfunction induced by OSI.

Oxidative stress results from an imbalance between oxidants and antioxidants within cells [4]; namely, the enhanced ROS overwhelms the antioxidative capacity and subsequently leads to endothelial dysfunction [34]. GSH-Px, SOD, and other enzymatic and nonenzymatic antioxidants play pivotal roles in preventing the cellular damage caused by ROS. In the present study, an obvious elevation of LDH and ROS production along with significant decrease in GSH-Px as well as SOD was observed in HUVECs after subjected to H_2_O_2_, indicating the shift of the balance between oxidative systems and antioxidant defenses. Nonetheless, when HUVECs were supplied with PD, the changes of these indexes induced by H_2_O_2_ were partially reversed. This is in agreement with previous reports confirming that administration of PD significantly limits LDH release from the I/R infracted myocardial tissue [14] and elevates the activity of SOD in I/R injured lung [10]. Similarly, PD has also been reported to exert antioxidative activity in kidney [8] and brain [9] as indicated before. These results clearly suggest that PD is a potent antioxidant against OSI related diseases.

Given that PD has antiapoptotic abilities [35, 36], we confirmed these findings of the change of several apoptosis related indexes including TUNEL staining positive ratio, caspase-3 activity, and Bax and Bcl-2 levels. Here, we found that the treatment with PD could significantly restrain H_2_O_2_-induced apoptosis, as verified by the reduction of TUNEL positive ratio, the caspase-3 activity, and Bax expression as well as by the upregulation of Bcl-2 expression. There is abundant evidence to support that PKC signaling plays a crucial role in OSI events. For instance, PKC is activated in redox stress in cardiac muscle cells [37] and endothelial cells [38]. As expected, the aforementioned antiapoptotic effect of PD vanished after PKC consistent activation by THX. Collectively, these results indicated that forced activation of PKC signaling could abolish the antiapoptotic effects of PD under oxidative stress condition. Our study demonstrated for the first time that PD may suppress apoptosis in H_2_O_2_-injured HUVECs through inhibiting PKC signaling pathway. These findings are in line with other studies, in which PD exerts protective effects against myocardial I/R and pulmonary hypertension through interaction with PKC signaling [14, 15].

Among PKC isoforms, PKCs *α*, *β*, *δ*, and *ε* are mainly involved in the pathogenesis of CVDs [20]. In the present study, conventional isoform *α*, novel isoforms *δ* and *ε*, and their phosphorylation were examined in HUVECs. PKC *α* has been validated to enhance superoxide production via NADPH oxidase [39] and to be activated by reactive oxygen species production in endothelial cells, which in turn activated the apoptosis pathway [40]. Upon activation, PKC *δ* mediates oxidative stress and cell death in a dietary model of nonalcoholic steatohepatitis [22] and in endothelia cells of lungs [41]. It is of special interest to investigate whether PD attenuates OSI in HUVECs through inhibition of PKCs *α* and *δ* cellular circuit. Our observation of significant phosphorylation of PKCs *α* and *δ* in HUVECs after exposure to H_2_O_2_ is consistent with the previous results obtained in COS-7 cells [30].

Meanwhile, as our results showed, the phosphorylation of PKCs *α* and *δ* decreased with PD replenishment. Interestingly, an enhanced 41 kDa proteolytic fragment below the total PKC *δ* protein band appeared when the cells were subjected to H_2_O_2_. This proteolytic fragment is probably caused by the elevated caspase-3 activity during H_2_O_2_ stimulation, which is consistent with other articles showing that caspase-3 cleaves the native PKC *δ* into a 41 kDa catalytically active fragment and a 38 kDa regulatory fragment to keep activating the kinase [42, 43]. The cleavage fragment has high catalytic activity and plays a key role in promoting apoptotic cell death. Thus, attenuation of PKC *δ* proteolytic activation may be also involved in the antiapoptotic effect of PD.

PKC *ε*, another member of novel PKC subfamily, is highly homologous to PKC *δ*. However, PKC *ε* and PKC *δ* sometimes have opposite effects. For example, PKC *ε* activation involved in isoflurane pretreatment could activate downstream signaling pathways and exert cardioprotection, which is directly opposed to that of PKC *δ* [24]. As shown in our results, there was little difference in PKC *ε* phosphorylation between the control group and H_2_O_2_-treated group, but the p-PKC *ε* significantly increased after being treated with PD. Similarly, previous research has shown that decrease in the apoptosis of cardiocytes damaged by H_2_O_2_ is partially attributed to the increased p-PKC *ε* [44]. Altogether, these studies reveal that PD protects H_2_O_2_-induced OSI through activation of PKC *ε*.

## 5. Conclusions

In summary, the present study demonstrates that PD could prevent HUVECs from H_2_O_2_-induced OSI, as evidenced by improvements of cell viability, adhesive and migratory ability, the rebalance between intracellular oxidants and antioxidants, and the inhibition of apoptosis. Nevertheless, the protective effects of PD are totally reversed when PKC signaling is compulsively activated by THX. Moreover, the phosphorylation of PKCs *α* and *δ* significantly decreases while the phosphorylation of PKC *ε* conspicuously increases after PD replenishment. Hence, PD may exert its antioxidative stress effects on H_2_O_2_-injured HUVECs through PKC dependent pathway.

## References

[1] Humbert M., Morrell N. W., Archer S. L. (2004). Cellular and molecular pathobiology of pulmonary arterial hypertension. *Journal of the American College of Cardiology*.

[2] Ross R. (1999). Atherosclerosis—an inflammatory disease. *New England Journal of Medicine*.

[3] de Berrazueta J. R., Guerra-Ruiz A., García-Unzueta M. T. (2010). Endothelial dysfunction, measured by reactive hyperaemia using strain-gauge plethysmography, is an independent predictor of adverse outcome in heart failure. *European Journal of Heart Failure*.

[4] Heitzer T., Schlinzig T., Krohn K., Meinertz T., Münzel T. (2001). Endothelial dysfunction, oxidative stress, and risk of cardiovascular events in patients with coronary artery disease. *Circulation*.

[5] Sinha K., Das J., Pal P. B., Sil P. C. (2013). Oxidative stress: the mitochondria-dependent and mitochondria-independent pathways of apoptosis. *Archives of Toxicology*.

[6] Zhang P. W., Yu C. L., Wang Y. Z. (1995). Influence of 3,4′,5-trihydroxystibene-3-beta-mono-D-glucoside on vascular endothelial epoprostenol and platelet aggregation. *Zhongguo Yao Li Xue Bao*.

[7] Du J., Sun L.-N., Xing W.-W. (2009). Lipid-lowering effects of polydatin from *Polygonum cuspidatum* in hyperlipidemic hamsters. *Phytomedicine*.

[8] Chen L., Lan Z., Lin Q. (2013). Polydatin ameliorates renal injury by attenuating oxidative stress-related inflammatory responses in fructose-induced urate nephropathic mice. *Food and Chemical Toxicology*.

[9] Cheng Y., Zhang H.-T., Sun L. (2006). Involvement of cell adhesion molecules in polydatin protection of brain tissues from ischemia-reperfusion injury. *Brain Research*.

[10] Wang F.-Y., Xu Z.-J., Zhang X.-L., Wang W.-T., Ha M.-L., Wang Y. (2008). Protective effects of polydatin against lung ischemia/reperfusion injury and the initial exploration for its mechanism. *Zhongguo Ying Yong Sheng Li Xue Za Zhi*.

[11] Zhang L. P., Ma H. J., Bu H. M. (2009). Polydatin attenuates ischemia/reperfusion-induced apoptosis in myocardium of the rat. *Sheng Li Xue Bao*.

[12] Hosoda R., Kuno A., Hori Y. S. (2013). Differential cell-protective function of two resveratrol (trans-3,5,4′-trihydroxystilbene) glucosides against oxidative stress. *Journal of Pharmacology and Experimental Therapeutics*.

[13] Sun D., Yue Q., Guo W W., et al (2015). Neuroprotection of resveratrol against neurotoxicity induced by methamphetamine in mouse mesencephalic dopaminergic neurons. *Biofactors*.

[14] Miao Q., Wang S., Miao S., Wang J., Xie Y., Yang Q. (2011). Cardioprotective effect of polydatin against ischemia/reperfusion injury: roles of protein kinase C and mito K_ATP_ activation. *Phytomedicine*.

[15] Miao Q., Shi X. P., Ye M. X. (2012). Polydatin attenuates hypoxic pulmonary hypertension and reverses remodeling through protein kinase C mechanisms. *International Journal of Molecular Sciences*.

[16] Nitti M., Furfaro A. L., Cevasco C. (2010). PKC delta and NADPH oxidase in retinoic acid-induced neuroblastoma cell differentiation. *Cellular Signalling*.

[17] Nakagawa M., Oliva J. L., Kothapalli D., Fournier A., Assoian R. K., Kazanietz M. G. (2005). Phorbol ester-induced G1 phase arrest selectively mediated by protein kinase C*δ*-dependent induction of p21. *The Journal of Biological Chemistry*.

[18] Chen J.-L., Lin H. H., Kim K.-J., Lin A., Ou J.-H. J., Ann D. K. (2009). PKC*δ* signaling: a dual role in regulating hypoxic stress-induced autophagy and apoptosis. *Autophagy*.

[19] Finkel T. (2003). Oxidant signals and oxidative stress. *Current Opinion in Cell Biology*.

[20] Steinberg S. F. (2012). Cardiac actions of protein kinase C isoforms. *Physiology*.

[21] Takeishi Y., Jalili T., Ball N. A., Walsh R. A. (1999). Responses of cardiac protein kinase C isoforms to distinct pathological stimuli are differentially regulated. *Circulation Research*.

[22] Greene M. W., Burrington C. M., Lynch D. T. (2014). Lipid metabolism, oxidative stress and cell death are regulated by PKC delta in a dietary model of nonalcoholic steatohepatitis. *PLoS ONE*.

[23] Xu H., Wang Q., Yang M., Yu J. (2015). 6B.03: tetrahydrobiopterin effects left ventricular diastolic function by upregulating protein kinase C epsilon signaling pathway in desoxycorticosterone acetate-salt hypertensive mice. *Journal of Hypertension*.

[24] Li H., Lang X.-E. (2015). Protein kinase C signaling pathway involvement in cardioprotection during isoflurane pretreatment. *Molecular Medicine Reports*.

[25] Liu H.-T., Li W.-M., Xu G. (2009). Chitosan oligosaccharides attenuate hydrogen peroxide-induced stress injury in human umbilical vein endothelial cells. *Pharmacological Research*.

[26] Huang P.-H., Chen J.-S., Tsai H.-Y. (2011). Globular adiponectin improves high glucose-suppressed endothelial progenitor cell function through endothelial nitric oxide synthase dependent mechanisms. *Journal of Molecular and Cellular Cardiology*.

[27] Cheong S.-M., Choi H., Hong B. S., Gho Y. S., Han J.-K. (2012). Dab2 is pivotal for endothelial cell migration by mediating VEGF expression in cancer cells. *Experimental Cell Research*.

[28] Altarescu G., Seror-Bukris O., Zimran A., Elstein D. (2010). Proteinase-activated receptor (PAR1) polymorphic variant correlates with thrombocytopenia in Gaucher disease. *Blood Cells, Molecules, and Diseases*.

[29] Visigalli R., Barilli A., Parolari A. (2010). Regulation of arginine transport and metabolism by Protein Kinase C*α* in endothelial cells: stimulation of CAT2 transporters and arginase activity. *Journal of Molecular and Cellular Cardiology*.

[30] Konishi H., Tanaka M., Takemura Y. (1997). Activation of protein kinase C by tyrosine phosphorylation in response to H_2_O_2_. *Proceedings of the National Academy of Sciences of the United States of America*.

[31] Boulden B. M., Widder J. D., Allen J. C. (2006). Early determinants of H_2_O_2_-induced endothelial dysfunction. *Free Radical Biology and Medicine*.

[32] Zhou D.-Y., Su Y., Gao P., Yang Q.-H., Wang Z., Xu Q. (2015). Resveratrol ameliorates high glucose-induced oxidative stress injury in human umbilical vein endothelial cells by activating AMPK. *Life Sciences*.

[33] Gu J., Wang C. Q., Fan H. H. (2006). Effects of resveratrol on endothelial progenitor cells and their contributions to reendothelialization in intima-injured rats. *Journal of Cardiovascular Pharmacology*.

[34] Cai H., Harrison D. G. (2000). Endothelial dysfunction in cardiovascular diseases: the role of oxidant stress. *Circulation Research*.

[35] Zeng Z., Chen Z., Li T., et al (2015). Polydatin: a new therapeutic agent against multiorgan dysfunction. *Journal of Surgical Research*.

[36] Li T., Liu Y., Li G. C. (2014). Polydatin attenuates ipopolysaccharide-induced acute lung injury in rats. *International Journal of Clinical and Experimental Pathology*.

[37] Rao F., Deng C. Y., Zhang Q. H. (2013). Involvement of Src tyrosine kinase and protein kinase C in the expression of macrophage migration inhibitory factor induced by H_2_O_2_ in HL-1 mouse cardiac muscle cells. *Brazilian Journal of Medical and Biological Research*.

[38] Gallo A., Ceolotto G., Pinton P. (2005). Metformin prevents glucose-induced protein kinase C-*β*2 activation in human umbilical vein endothelial cells through an antioxidant mechanism. *Diabetes*.

[39] Herrera M., Silva G. B., Garvin J. L. (2010). Angiotensin II stimulates thick ascending limb superoxide production via protein kinase C*α*-dependent NADPH oxidase activation. *The Journal of Biological Chemistry*.

[40] Hecquet C. M., Zhang M., Mittal M. (2014). Cooperative interaction of trp melastatin channel transient receptor potential (TRPM2) with its splice variant TRPM2 short variant is essential for endothelial cell apoptosis. *Circulation Research*.

[41] Grinnell K., Duong H., Newton J., Rounds S., Choudhary G., Harrington E. O. (2012). Heterogeneity in apoptotic responses of microvascular endothelial cells to oxidative stress. *Journal of Cellular Physiology*.

[42] Kanthasamy A. G., Kitazawa M., Kanthasamy A., Anantharam V. (2003). Role of proteolytic activation of protein kinase C*δ* in oxidative stress-induced apoptosis. *Antioxidants & Redox Signaling*.

[43] Kato K., Yamanouchi D., Esbona K. (2009). Caspase-mediated protein kinase C-delta cleavage is necessary for apoptosis of vascular smooth muscle cells. *American Journal of Physiology—Heart and Circulatory Physiology*.

[44] Wang S. S., Ji Y. S., Li H., Yang S. J. (2009). Mechanisms of gross saponins of tribulus terrestris via activating PKC*ε* against myocardial apoptosis induced by oxidative stress. *Yao Xue Xue Bao*.

